# Sustainable Drying and Green Deep Eutectic Extraction of Carotenoids from Tomato Pomace

**DOI:** 10.3390/foods11030405

**Published:** 2022-01-30

**Authors:** Celeste Lazzarini, Enrico Casadei, Enrico Valli, Matilde Tura, Luigi Ragni, Alessandra Bendini, Tullia Gallina Toschi

**Affiliations:** 1 Department of Agricultural and Food Sciences (DISTAL), Alma Mater Studiorum—Università di Bologna, Piazza Goidanich 60, 47521 Cesena, Italy; celeste.lazzarini3@unibo.it (C.L.); enrico.casadei15@unibo.it (E.C.); luigi.ragni@unibo.it (L.R.); alessandra.bendini@unibo.it (A.B.); 2 Interdepartmental Centre for Industrial Agrofood Research (CIRI Agroalimentare), Alma Mater Studiorum—Università di Bologna, Via Quinto Bucci 336, 47521 Cesena, Italy; tullia.gallinatoschi@unibo.it; 3 Department of Agricultural and Food Sciences (DISTAL), Alma Mater Studiorum—Università di Bologna, Viale Fanin, 40, 40127 Bologna, Italy; matilde.tura2@unibo.it

**Keywords:** tomato pomace, lycopene, β-carotene, extraction, sustainability, food by-products, deep eutectic solvents, non-thermal drying

## Abstract

The extraction of molecules with high added value plays an important role in the recovery of food waste. This work aimed to valorize tomato pomace, a by-product composed of skin and seeds, through extraction of carotenoids, especially lycopene and β-carotene. The tomato pomace was dried using three different methods (freeze-drying, heat drying, and non-thermal air-drying) to reduce its weight, volume, and water activity and to concentrate the carotenoid fraction. These drying approaches were compared considering the extractive potential. Three solvent mixtures were compared, a traditional one (*n*-hexane:acetone) and two green deep eutectic solvent mixtures (ethyl acetate:ethyl lactate and menthol:lactic acid) in combination with different drying procedures. The extract obtained using ethyl acetate:ethyl lactate with non-thermal air-drying showed the highest contents of lycopene and β-carotene (75.86 and 3950.08 µg/g of dried sample, respectively) compared with the other procedures.

## 1. Introduction

The cultivated tomato, *Solanum lycopersicum* L., is the world’s most highly consumed vegetable, thanks to its versatility and its status as an ingredient in a large variety of different foods [1]. In 2018, it was estimated that nearly 5 billion hectares are cultivated to produce tomatoes, with an average production of more than 182 billion tons worldwide [2]. 

Tomato skin and seeds are usually undesirable parts for the preparation of most tomato derivates, and thus there is the need for separation from the pulp. The produced by-product accounts for 1.5 to 5% of the initial weight of the fruit, which is a concerning amount of material considering its widespread cultivation [3,4,5,6]. This tomato by-product, called tomato pomace, is rich in fiber and other important compounds such as sugars, proteins, pectins, fats, and vitamins [3]. The peel has a higher content of fibers, carotenoids, and phenols than seeds, which mainly consist of oil and proteins [4,7,8,9,10]. 

Tomatoes and tomato products meet consumer demands in terms of cost, convenience, availability, and taste, while they also deliver beneficial health effects, being easily included in a large variety of culturally diverse dishes [11]. Carotenoids are naturally present in tomatoes; among these is lycopene, which is mainly responsible for the red color of the fruit with a claimed nutraceutical effect [12], and it also appears to act as a powerful antioxidant, preventing the action of free radicals and carcinogenic cells [13,14]. Tomato can play an important role in valorization of by-products, not only for feedstock and production of biofuels [15,16,17], but also through the extraction of important compounds, including lycopene [18]. In fact, lycopene can be used in cosmetic preparations due to its properties of reducing lipid oxidation and preventing damage related to the action of UV rays [18]. The antioxidant activity of carotenoids can also be applied to the food supplement field [19] and in the meat industry against lipid oxidation and as colorants, thus improving the oxidative stability of meat products [20]. Indeed, natural antioxidants are safe to be consumed and can be generally obtained with fewer rough chemical processes rather than synthetic ones; they can be extracted and applied in different food sectors in addition to the meat industry [21]. The food industry nowadays prefers “green” solvents for the extraction of these compounds, due to their non-toxic features, food safety, and recycling possibilities [22].

Moreover, colors play an important role in the marketing success of any food product and can often influence consumer preferences [23,24]; following the trend of more interest in natural products, even natural colorants are preferred as a healthier alternative to synthetic ones [25]. 

Lycopene is responsible for the bright reddish color of tomato and tomato-based products: its color is due to its chemical characteristics and its eleven linear conjugated double bonds in a polyene chain, which is able to absorb almost all visible light radiation while reflecting low-frequency wavelengths [26]. Due to its strong shade and absence of toxicity, this pigment is used for a wide range of applications in food industries [27]. 

β-carotene is also an important natural colorant, which is responsible for the orange color of tomato and other vegetables such as carrots [28]; natural pigments are responsible for the appearance of fruits and vegetables and for their visual attractiveness, and their consumption is associated with health benefits, such as the decreased risk of developing of some diseases [28,29].

Lycopene has gained increasing importance thanks to its unique properties; in this context, the extraction of carotenoids is also fundamental to take advantage of their full potential [30]. According to FAOSTAT [31], the total production of food as the major source of lycopene (namely tomatoes, pumpkins, and melons) has increased annually as has the associated waste production, which are usually lycopene-rich parts [32]. The global nutraceutical market reached a value of USD 289.8 billion in 2021, including 1.5 billion for carotenoids in 2017 and USD 2.0 billion in 2022 [33,34], while lycopene accounts for about 7% of the total market [35]. 

β-carotene has also gained increasing importance thanks to its antioxidant properties [36]; it also has other interesting properties such as protection against cardiovascular disease and cancer, improvement of the immune system, filtration of UV-light, and anti-inflammatory activities [37]. 

As for lycopene, β-carotene has also seen a major demand, reaching high prices (more than 500 EUR per kg), making tomato pomace a profitable source of this important biological compound [38,39]. Moreover, traditional solvent extraction may have low efficiency with consumption of large amounts of solvent and time [40,41,42]; moreover, the solvents may be harmful for both the environment and human health [43,44]. Organic solvents can easily enter body parts and organs, where they are converted via osmotic processes into water-soluble forms, which sometimes can be more toxic than the parent compound [45]. 

Organic solvents are highly volatile and are more likely to be inhaled via respiration, affecting the lungs and other organs of operators; therefore, replacement with greener and less toxic solvents is becoming increasingly important due to increasing health and environmental concerns [45,46]. Moreover, new emerging technologies, such as ultrasound-assisted extraction, can promote lycopene extraction while preserving this important compound for human and environmental health [32]. 

In particular, to avoid the potentially deleterious effects of traditional solvents, new classes of reagents have become more popular, such as deep eutectic solvents (DES), a particular class of solvents considered to be environmentally friendly [47]. A DES mixture is made of two or more compounds that are typically solid at room temperature, but when mixed at particular ratios, change into a liquid [48]. DES can be easily synthesized by combining a hydrogen bond acceptor (HBA) and a hydrogen bond donor (HBD), and their combination results in changes in the physical characteristics of the mixture (e.g., melting point, solubility) [47]. 

Therefore, their biodegradability is extraordinarily high, and their toxicity is nonexistent or very low. Because of their minimal ecological footprint, low cost of their constituents, tunability of their physicochemical properties, and ease of preparation, DESs are successfully and progressively replacing often hazardous and volatile organic compounds (VOCs) in many fields of science [49]. 

DESs have several properties that make them suitable for different types of extraction: they are not derived from petroleum, while they are cheap, easy to prepare, and biodegradable and have high purity [50]. In addition, one of the major advantages related to the use of DESs is their tunability: the physical characteristics of a large number of eutectic mixtures can be varied (such as viscosity, density, etc.) by simply changing one or more of the components of the mixture [47]. 

Concerning green extraction, a variety of techniques can be considered, one of which is supercritical fluid extraction (SFE). This technique is increasingly used, thanks to its versatility that allows one to fine-tune the solvent; this can be done by modifying its polarity, e.g., with ethanol, according to the polarity of the target compounds. Moreover, when using CO_2_, since it is volatile at ambient conditions, it can be easily separated from the extract, with benefits for health and the environment [51]. The use of supercritical CO_2_ can successfully extract thermolabile compounds (generally at temperatures around 60 °C), such as carotenoids, considering the inert characteristics of this solvent, namely non-explosive and non-toxic characteristics [51]. Technologies such as supercritical fluid extraction require particular equipment that is comprehensive of pumps, stainless steel extraction vessels, pipes, etc., and therefore upscaling of this technology would be very expensive. However, once the facilities are present, the required solvent is easily available and inexpensive [52]. Generally, due to high costs, this technique is frequently used to extract valuable compounds such as the those used in the food, pharmaceutical, and cosmetic industries [52].

Drying methods are fundamental to enhance extraction yields and improve durability, especially considering the high water content of by-products typical of the food industry; one of the most common is freeze-drying, which is one of the best techniques to prevent the spoilage of samples and to preserve composition [53]. Even if freeze-drying is able to drastically reduce the water content of dried material, it requires large amounts of energy and is also expensive, especially depending on the material to be dried; it has been estimated that the cost of freeze-drying is nearly eight times higher than the cost of air drying, which can be applied for high-value products and compounds [54,55].

Other drying techniques involve the use of heat, such as oven drying, which involves exposure to high temperatures with adverse effects on the nutritional and chemical properties [56]. The use of an innovative prototype of non-thermal air-drying has the potential to overcome the drawbacks related to the use of thermal energy. 

This research tested three different solvent mixtures on a tomato pomace composed of peels and seeds, comparing their extractive potential in terms of lycopene and β-carotene content. Furthermore, three drying methods, namely freeze-drying, heat drying in a conventional oven, and nonthermal air-drying with the use of a prototype, were applied to favor the action of the solvents used. This study thus investigated the possible substitutions of traditional extraction techniques and drying methods with more sustainable ones with comparable extractive potentials, which is an essential aspect for any industrial application. 

## 2. Materials and Methods

The raw material used was tomato pomace, made of skins and seeds, from the industrial production of tomato purée. After collection—performed in an Italian company, named La Cesenate Conserve Alimentari S.p.a. located in Cesena, immediately after production of the purée—pomace was stored, packed in plastic bags containing 5 kg each, in a freezer at −40 °C until extraction tests in order to preserve its characteristics. 

### 2.1. Water Content and Water Activity (a_w_)

Preliminary analyses of the physical characteristics of the tomato by-product were performed. To quantify the water content, a gravimetric method was used by weighing 5 g of tomato pomace and drying it with the use of a traditional lab oven (M20-VN, PID system, Monza Brianza, Italy) at 105 °C. The samples were kept in the oven until their weight was stable (around 8 h), and the analysis was carried out in three replicates.

The water content was calculated with the following equation:

(1) $$Water\;content\;\%=\frac{m_{h}-m_{d}}{m_{h}}100$$

where *m_h_* refers to the weight of the humid tomato pomace and *m_d_* of the dried material. 

For a_w_, the analysis was done after defrosting at 20 °C the tomato pomace in specific plastic containers, placing the product on the bottom of the disk in such a way to cover its entire surface, and quantified using Aqualab (Series 3, Decagon Devices Inc., Pullman, WA, USA).

Both water content and water activity analyses were repeated in three replicates for the raw tomato pomace and on each dried by-product. 

### 2.2. Drying Treatments

To enhance the effectiveness of the solvent mixtures to extract carotenoids from the by-product (see Section 2.4), the following procedures were used. For (lab-scale) freeze-drying (Heto PowerDry LL3000 Freeze Dryer, Thermo Fisher Scientifics, Warminster, United Kingdom), the by-product was stored for 12 h at −40 °C and was then freeze-dried within a 27 h treatment cycle (the samples were coded as “L”). For air-drying treatment (sample code “E”), a prototype previously developed at Interdepartmental Centre for Industrial Agrofood Research (CIRI Agro, Cesena, Italy) that does not use thermal energy was used, thus preserving the by-product from degradation. The prototype is composed of a pseudo stadium-shaped chamber in which the material is set. The movement of a blade rotor, together with a flux of compressed air, allows ventilation, closed-loop circulation, and crunching of the tomato pomace. The overall external dimensions of the chamber, made of stainless steel, are 80 cm × 50 cm, with a height of 25 cm, while the four-blade helical rotor has a diameter of 24 cm. It is rotated by a 0.37 kW three-phase asynchronous motor. The rotation speed can be regulated using an inverter up to its maximum nominal value of 2790 min^−1^. The compressed air flow is injected tangentially to promote circulation of the air-product flow. The drying procedure took 2.5 h, in which the by-product was mixed to guarantee homogeneity every 30 min.

The procedure was also carried out through a traditional heating laboratory oven (PID system M20-VN) (sample code “S”) set at 85 °C, chosen as a temperature to preserve as much as possible the characteristics of tomato pomace based on the literature [57]. To reach a comparable a_w_ with respect to the air-dried material, the process took 2 h. 

### 2.3. Carotenoids Extraction Methods

The extraction methods described herein use different categories of solvents, both traditional and green. For traditional solvents, an *n*-hexane-acetone (1:1 % *v*/*v*) (*n*-hexane purchased from Sigma Aldrich, with purity ≥97%, Darmstadt, Germany and acetone purchased from Carlo Erba Reagents, with purity ≥99.8%, Cornaredo, Milan, Italy) mixture was tested (the extracts were coded as “T”), while DES DL-menthol and lactic acid solution (1:8 molar ratio) (code “ML”) (DL menthol from Sigma Aldrich, purity ≥98%, Darmstadt, Germany, and lactic acid Sigma Aldrich, purity ≥85 %, Darmstadt, Germany), ethyl acetate (Sigma Aldrich, purity ≥99.8%, Darmstadt, Germany), and ethyl lactate (Sigma Aldrich, purity ≥98%, Darmstadt, Germany) solution (30% of ethyl acetate and 70% of ethyl lactate) (code “G”) were used as the DES solvents. The procedure follows previously developed analytical protocols and applied in the literature on the same matrix [50,58]. 

To compare the three extraction procedures, it is fundamental to adopt the same conditions in terms of temperature, time, and solvent/sample volume/mass ratio: according to the literature, the parameters that favor carotenoid extraction are 63 °C for 20 min in ultrasonic bath (2209 S3 Ultrasonic Cleaner, Milan, Italy), with 100 mL/mg solvent/sample, both for *n*-hexane:acetone solution and ethyl acetate:ethyl lactate, but 120 mg/L for the DL-menthol:lactic acid solution [50,58]. As reported by Silva and colleagues [50,58], the solvents were prepared following the following ratios: 1:1 % *v/v n*-hexane:acetone, 70:30 *v/v* ethyl acetate:ethyl lactate, 8:1 mol/mol DL-menthol:lactic acid. 

After preparation of the three above-mentioned solvent mixtures, 0.2 g of raw by-product was weighed for each sample, doubling the quantities suggested by the operation procedures tested by Silva and colleagues [50,58] due to the high moisture content of tomato pomace. When the extraction was performed on treated by-product (freeze-dried, air-dried and heat-dried), 0.1 g of by-product was considered. Next, 10 mL of *n*-hexane:acetone was added to a test tube for a trial, 10 mL of ethyl acetate:ethyl lactate for another, and lastly 12 mL of DL-menthol:lactic acid for another, in three replicates each; each solvent mixture was heated at 60 °C before the interaction with the by-product. After this pre-heating, the solvent mixture was mixed with the tomato pomace using a vortex (Vortex agitator Zx3, VELP Scientifica Srl, Monza Brianza, Italy) for 10 s and placed in an ultrasound water bath at 60 °C (±1 °C) for 20 min. 

Finally, the glass tubes were removed from the water bath and centrifuged (MPW M-SCIENCE, MPW Med. Instruments, Warsaw, Poland) at 2916 rpm for 10 min to obtain the separation of the solid and liquid phases. The liquid extract was withdrawn and transferred into another glass tube for spectrophotometric analyses. 

### 2.4. Spectrophotometric Analyses

The quantification of carotenoid content was performed by spectrophotometric analysis (spectrophotometer UV-1800, Shimadzu, Kyoto, Japan) using specific calibration curves for lycopene (Supelco, purity ≥90% Darmstadt, Germany) and β-carotene (Sigma Aldrich, purity ≥97%, Darmstadt, Germany) constructed by dissolving the related standards at different concentrations in dichloromethane solutions. Dichloromethane (λ cut-off ≤ 233 nm) (Sigma Aldrich, purity ≥99.9%, Darmstadt, Germany) was chosen because it can dissolve both carotenoids; moreover, preliminary tests were performed to check the spectra between 250 and 600 nm of lycopene and β-carotene solutions dissolved in dichloromethane and in the other solvents (*n*-hexane/acetone, ethyl acetate/ethyl lactate and menthol/lactic acid). The resulting peaks were very similar for each solution. Absorbance peaks were identified for lycopene at 477 nm and 481 nm, and for β-carotene at 461 nm. Both wavelengths were considered for lycopene (477 nm for the extractions using the green solvent solutions according to the literature [58] and 481 nm for the extracts obtained with the conventional one according to the absorbance peak obtained from the traditional extract). The calibration curve of lycopene at 481 nm has the equation y = 0.2481x (R^2^ = 0.9957), the one at 477 nm y = 0.2393x (R^2^ = 0.9829), and the curve of β-carotene y = 0.0042x + 0.0265 (R^2^ = 0.9928). 

The absorbance of each extract was determined using a UV-spectrophotometer at the abovementioned wavelengths against the respective blank (the different solvent mixtures) and linear regression equations (R^2^ > 0.99) were obtained for the three calibration curves and used to determine the lycopene and β-carotene content (µg/g) in each extract; the absorbance of the solvent (blank) concentrations was expressed as µg carotenoids per g of by-product weighted. A regular check was carried out for the accuracy and reproducibility of the absorbance and wavelength scales as well as for stray light.

Color of the extracts was also determined. A quartz cuvette was filled with the extract, and the transmittance was read on a Jasco dual beam spectrophotometer model V-550 UV-Vis (Jasco, Tokyo, Japan). Results were expressed using the CIEL*a*b* scale.

### 2.5. Data Processing and Statistical Analysis 

Data processing and calculation were carried out with Microsoft^®^ spreadsheet program 2016 (Microsoft Corp., Redmond, WA, USA). Analysis of variance (Analysis of variance (one-way ANOVA, Tukey’s HSD, *p* < 0.05) and PCA were performed with XLSTAT (Addinsoft Corp., Paris, France).

## 3. Results and Discussion

### 3.1. Water Content and Water Activity (a_w_) in the Raw and Treated By-Products

The analyses of moisture level and water activity are relevant to assess how each treatment described in Section 2.3 can preserve the by-product by drying it out. As seen in Table 1, the initial moisture content was 63% while the water activity was 0.99. The freeze-dried by-product had the lowest level of moisture content and a_w_, followed by the non–heat-dried by-product obtained with the prototype. This highlights the satisfactory action of the air-drying prototype, since it allows the level of a_w_ to decrease below 0.7, while the lowest level of a_w_ was reached with the freeze-drier. According to the literature, at an a_w_ lower than 0.75, bacterial growth is inhibited [59]. Finally, the heat-dried by-product had a_w_ values comparable to those obtained with the prototype, thus supporting possible inhibition of bacterial activity despite the higher moisture content observed for this treatment compared to the two other drying methods; these results are similar to those found in the literature for comparable products [60]. 

### 3.2. Lycopene and β-Carotene Content in the Extracts

The carotenoid content in extracts strongly differ in relation to both the solvent used and the drying method (Table 2).

Concerning the solvent mixture, the one with the highest extraction potential was the traditional one, acetone:*n*-hexane, which had some practical issues related to evaporation of the solvent during the heating/ultrasound-assisted extraction. This could be useful in a closed system, with possible solvent recovery and extract concentration, but in an open system, it is not sustainable. The use of the DES mixture, composed of ethyl acetate:ethyl lactate, showed promising results in terms of amount of lycopene extracted. In fact, the DES eutectic mixture (samples with code “G”) was the second-best option for extraction of carotenoids from the raw by-product, and in particular lycopene, compared to the ultrasound-assisted extraction conducted with the use of traditional solvents (code “T” in Table 2).

In fact, from the raw by-product treated with the greener solvent solution, it is possible to obtain extracts with 27.44 µg/g of lycopene (sample G), which is significantly lower than the 34.11 µg/g obtained with acetone:*n*-hexane (sample T). The use of DES allows practical issues to be overcome related to the high volatility of the traditional organic solution. A similar result was seen for β-carotene with 1510.19 µg/g (sample G) obtained with ethyl acetate:ethyl lactate compared with 2117.64 µg/g from the traditional extraction (sample T). 

On the other hand, the DL-menthol:lactic acid solution showed difficulties in the interaction between the solvents in the mixture and the by-product due to its viscosity, thus lowering the extraction potential, with 12.32 µg/g and 492.46 µg/g, respectively, for lycopene and β-carotene (sample ML). 

Moreover, on the dried by-product, both the drying method and the choice of the extraction mixture had a strong influence on the lycopene and β-carotene content in the final extracts (Table 2). In particular, concerning the drying methods, the prototype (E) showed good potential while preserving the carotenoid content, as shown in Table 3. The use of non-thermal air-drying was superior to freeze-drying in terms of extractions with the DES mixture for both lycopene and β-carotene. The nature of the prototype allows the by-product to be pulverized, and this appears extremely useful, rendering the subsequent interaction between solvent and smashed by-product more effective. Indeed, by reducing the particle size of the raw material, it is possible to enhance the extraction of lycopene and β-carotene; in fact, according to the literature, the extraction potential is inversely proportional to the particles dimension [61]. Indeed, the concentration of lycopene in the extracts obtained from the three different solvent mixtures of the by-product dried with the use of the prototype are comparable: (i) 81.20 µg/g with acetone:*n*-hexane (sample “TE”), (ii) 75.86 µg/g with ethyl acetate:ethyl lactate (sample “GE”), and (iii) 82.86 µg/g with menthol:lactic acid (sample MLE) (Table 2). These results confirm the high potential of the unconventional non-thermal drying procedure. In fact, it allows the by-product to be uniformly pulverized, thus enhancing the extraction potential, thanks to the synergic action of the flux of air and the rotation of the blade rotor, while preserving the most important biological compounds. For this reason, with non-thermal air-drying, the greener solvents performed at their best in terms of extractive capacity. 

The lycopene concentration of the by-product obviously has a decisive influence on the yield. In fact, Silva and colleagues [58], adopting the same extractive conditions, reported a higher lycopene content (1334.8 μg/g of dried material). Different storage conditions of the by-product in the factory before sampling and diverse tomato variety appear to be responsible for this variability. To verify if it is convenient to extract carotenoids and lycopene from a by-product of the tomato canning industry, the starting point is an evaluation of the quality and quantity of these components in the by-product itself. In addition, the lycopene content in extracts is lower than that of β-carotene; this is due to the previously mentioned storage and processing conditions that can lead to the degradation of lycopene, since its degradation rate is higher than that of β-carotene [62].

Concerning heat drying with the use of the oven (samples coded with “S”), the tests performed show that the lycopene and β-carotene content in the extracts were significantly lower than those found when applying the other drying treatments (Table 2). The main cause may be temperature, which is responsible for the degradation of carotenoids, and, in particular, the total decay of these compounds occurs at 100–145 °C according to the literature, even if a proportion is also affected by the increase in temperature, up to 80 °C for 2 h, used in the oven [57]. It is clear that heat treatments should be avoided when tomato pomace is used, independently of the solvent used for the subsequent extraction, since the carotenoid yield is dramatically reduced (Table 2). 

### 3.3. Color and Principal Component Analysis

The treatment to dry out the by-product and the choice of solvent mixture also showed a significant impact on the color of the extracts, in terms of L*a*b* (Table 3). 

**Figure 1:**
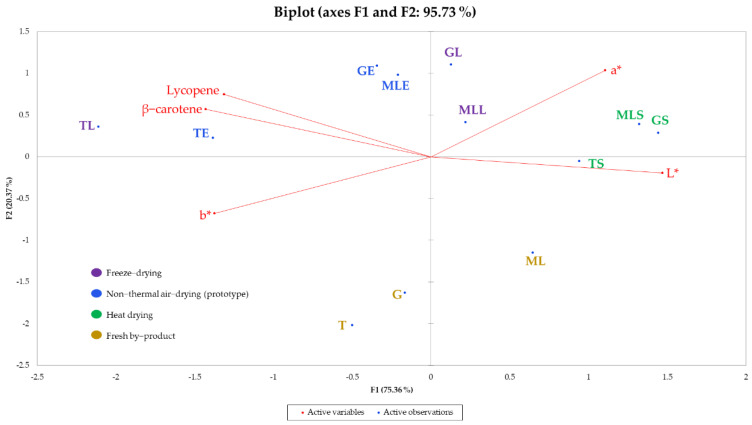
PCA biplot built with the results of the color analysis and the lycopene and β-carotene content for all the samples under consideration.

Based on some considerations, in terms of quality, explained in Section 3.2, the different extracts are clearly discriminated in a Principal Component Analysis (PCA) biplot (Figure 1), which takes into account, besides the determination of lycopene and β-carotene contents, the results of color determination.

As shown in Figure 1, the lycopene and β-carotene contents were negatively correlated with the L* (r = −0.807 and r = −0.905, respectively, *p* ≤ 0.05). On the other hand, b* was positively correlated with the β-carotene content (r = 0.651, *p* ≤ 0.05). The variable b*, if positive, is considered as a yellow index [63]. Extracts obtained from freeze-dried and non-thermal air-dried tomato pomace showed a higher level of carotenoids vs. fresh and heat dried (see Table 2) and higher percentages of the yellow and red components (see Table 3). The differences in color among the samples, also related to the content of lycopene and β-carotene, should be taken into consideration for the formulation of different cosmetic, food, and pharmaceutical products, such as the type of the solvent used for the extraction (e.g., food grade), its residual amount in a completely dried extract, or other possible restrictions through laws.

## 4. Conclusions

This study demonstrates advancements regarding the action of green solvents on food by-products, in combination with the use of an innovative non-thermal drying method. The results, in particular the lycopene extraction from the non–thermal-dried tomato by-product, confirm ethyl acetate:ethyl lactate mixture as an effective alternative to the traditional solvents. 

Regarding the drying methods tested, it is difficult to compare the differences in energy consumption, since the non-thermal drying prototype was produced only at a lab scale. However, its upgrade to an industrial or semi-industrial plant would surely bring significant advantages in terms of sustainability, compared to heating, which is typical of traditional dryers, or long-time freezing at low temperatures for freeze-drying, both of which require high energy consumption. This preliminary work has highlighted the possibility of drying and the potential of non-conventional extraction techniques to extract carotenoids; the use of HPLC to quantify them would be needed in further studies to confirm these findings. 

Furthermore, the synergy between a sustainable drying process and the green solvent used for the extraction appears to be a valid strategy to reduce energy consumption and, at the same time, sustain the environment.

In particular, for the tomato by-product and lycopene and β-carotene extraction, the valorization can be part of a project of industrial symbiosis, where the two technological phases—(i) concentration/stabilization and possible packaging of the by-product to guarantee a defined shelf-life and preserve it from spoilage and degradation and (ii) extraction of the fractions/molecules of interest—could be held in two different factories or even industrial chains. The first (stabilization/packaging) could become a new final phase of the tomato supply chain, and the second (extraction) could be conducted elsewhere in a biorefinery or in a specific industrial environment, putting in place tailored flux diagrams for cosmetic, food supplement, and/or pharmaceutical applications.

## Figures and Tables

**Table 1:** Mean (±SD) moisture content (%) and mean a_w_ for each tomato by-product, both raw and treated, as described in Section 2.3. By-product: raw (P), freeze-dried (L), non-thermal air-dried (E), and heat-dried (S).

Sample	Mean Moisture Content% ± SD	Mean a_w_
Raw (P)	63.4 ± 1.7	0.99
Freeze-dried (L)	3.5 ± 0.1	0.30
Air-dried (E)	9.9 ± 0.2	0.67
Heat-dried (S)	20.3 ± 0.2	0.69

**Table 2:** Carotenoid content in the extract obtained with the three different solvent solutions in combination with the three drying methods. The samples were named using the combination of codes, identifying the different solvent solution, acetone:*n*-hexane (1:1 % *v*/*v*) (T), ethyl acetate:ethyl lactate (30:70 %*v*/*v*) (G), and DL-menthol:lactic acid (8:1 mol/mol) (ML), and the three drying techniques, freeze-drying (L), non-thermal air-drying (E), and heat drying (S) (e.g., MLL: extract obtained from the use of DL-menthol:lactic acid from the freeze-dried by-product).

Extract	Lycopene Content (µg/g) ± SD	β-Carotene Content (µg/g) ± SD
T	34.11 ^a;B^ ± 1.11	2117.64 ^a;B^ ± 100.46
G	27.44 ^b;C^ ± 2.50	1510.19 ^b;B^ ± 79.51
ML	12.32 ^c;C^ ± 1.78	492.46 ^c;B^ ± 87.88
TL	96.55 ^a;A^ ± 10.39	5717.46 ^a;A^ ± 710.70
GL	56.28 ^b;B^ ± 6.07	3000.45 ^b;B,C^ ± 26.58
MLL	48.89 ^b;B^ ± 1.89	2273.87 ^b;A^ ± 92.58
TE	81.20 ^a;A^ ± 4.20	4777.59 ^a;A^ ± 256.04
GE	75.86 ^a;A^ ± 10.94	3950.08 ^a,b;A^ ± 597.49
MLE	82.86 ^a;A^ ± 11.18	2923.02 ^b;A^± 583.24
TS	15.87 ^a;B^ ± 0.21	507.29 ^a;C^ ± 48.56
GS	1.80 ^c;D^ ± 0.83	ND
MLS	11.22 ^b;C^ ± 0.45	78.27 ^b;B^ ± 21.61
Solvent mixture	<0.0001	<0.0001
Drying method (treatment)	<0.0001	<0.0001
Solvent mixture * Drying method (treatment)	<0.0001	<0.0001

Different lowercase letters indicate significant differences (one-way ANOVA, Tukey’s HSD, *p* < 0.05) among the extracts obtained by the raw and differently treated by-products using the same solvent mixtures. Different capital letters indicate significant differences (one-way ANOVA, Tukey’s HSD, *p* < 0.05) among the extracts, obtained by the raw and the treated by-product with the same drying technology, or non-treatment, using different solvent mixtures. * ND: not detected.

**Table 3:** CIE coordinates of the color space regarding the different samples of tomato by-product extracts. L* indicated the lightness, a* the red/green coordinate, and b* the yellow/blue. Higher values of a* are redder, while higher values of b are more yellow rather than blue.

Sample	a*	b*	L*
T	−6.01 ^b;C^	22.28 ^a;A^	95.31 ^b;A^
TL	−4.85 ^b;B,C^	25.85 ^a;A^	91.82 ^b;B^
TE	−4.32 ^b;B^	22.48 ^a;A^	93.43 ^a;B^
TS	−2.22 ^a;A^	7.91 ^a;B^	96.20 ^a;A^
G	−5.17 ^a,b;C^	18.74 ^a;A^	95.23 ^b;A^
GL	−2.32 ^a;A,B^	7.72 ^b;B,C^	95.27 ^a;A^
GE	−3.17 ^a;B^	9.73 ^b;B^	95.14 ^a;A^
GS	−1.34 ^a;A^	3.10 ^b;C^	95.51 ^a;A^
ML	−4.16 ^a;C^	10.42 ^b;A^	96.23 ^a;A^
MLL	−3.00 ^a;B^	9.17 ^b;A^	95.66 ^a;B^
MLE	−3.15 ^a;B^	9.71 ^b;A^	95.35 ^a;B^
MLS	−1.38 ^a;A^	3.86 ^b;B^	96.44 ^a;A^
Solvent mixture	<0.0001	<0.0001	<0.0001
Drying method (treatment)	<0.0001	<0.0001	<0.0001
Solvent mixture * Drying method (treatment)	<0.0001	0.005	0.010

Different lowercase letters indicate significant differences (one-way ANOVA, Tukey’s HSD, *p* < 0.05) among the extracts obtained by the raw and the differently treated by-products using the same solvent mixtures. Different capital letters indicate significant differences among the extracts, obtained by the raw and the treated by-product with the same drying technology using different solvent mixtures. * ND: not detected.

## Data Availability

Data is contained within the article.
